# Towards Machine Vision for Insect Welfare Monitoring and Behavioural Insights

**DOI:** 10.3389/fvets.2022.835529

**Published:** 2022-02-15

**Authors:** Mark F. Hansen, Alphonsus Oparaeke, Ryan Gallagher, Amir Karimi, Fahim Tariq, Melvyn L. Smith

**Affiliations:** ^1^The Centre for Machine Vision, Bristol Robotics Laboratory, UWE Bristol, Bristol, United Kingdom; ^2^Department of Crop Science, University of Calabar, Calabar, Nigeria; ^3^SciFlair Ltd., Bristol, United Kingdom

**Keywords:** machine vision, deep learning, insect farming, black soldier fly, domestic crickets, sex classification

## Abstract

Machine vision has demonstrated its usefulness in the livestock industry in terms of improving welfare in such areas as lameness detection and body condition scoring in dairy cattle. In this article, we present some promising results of applying state of the art object detection and classification techniques to insects, specifically Black Soldier Fly (BSF) and the domestic cricket, with the view of enabling automated processing for insect farming. We also present the low-cost “Insecto” Internet of Things (IoT) device, which provides environmental condition monitoring for temperature, humidity, CO_2_, air pressure, and volatile organic compound levels together with high resolution image capture. We show that we are able to accurately count and measure size of BSF larvae and also classify the sex of domestic crickets by detecting the presence of the ovipositor. These early results point to future work for enabling automation in the selection of desirable phenotypes for subsequent generations and for providing early alerts should environmental conditions deviate from desired values.

## 1. Introduction

Insects are currently a component in the diets of two billion people around the world (1) and 2,111 known insect species have been recorded as being consumed by people (2). There is a good reason for this. Insects such as crickets and BSF, like other types of arthropod, are remarkably efficient in fixing biomass that is highly protein rich; they are also hardy and easy to breed (3), require little or no processing before consuming, and they have a relatively short growth cycle. This makes them attractive for breeding in regions of the world which suffer from food insecurity problems that affect food supplies for both livestock and human consumption as well as offering potential alternative protein sources for livestock feed *via* waste processing in mode developed areas.

Conversely, when we consider the issue of food insecurity, food waste is a financial, humanitarian, and environmental concern, demanding a sustainable and efficient solution to manage the ever-increasing volume of nutrition loss. Naturally, some insects feed on organic waste and turn it into biomass. They are able to consume low-grade organic waste and convert it into usable bio-products such as animal protein and lipids (4), which could feed livestock animals, such as fish, poultry, and pigs.

For these reasons alone, insect farming has attracted considerable interest from industry as well as within academia, and this interest is predicted to increase over the next decade. Evidence for the commercial validity of insect farming is that there are already multiple companies and startups around the UK, such as Entocycle, Better Origin, and Beta Bugs, who are professionally farming insects like Black Soldier Fly. However, this approach is not limited to the UK and has been used across the globe, e.g., the USA, Southeast Asian countries such as Indonesia and Thailand as well as many countries in Africa.

Insect farmers require knowledge of the growing conditions within each growing enclosure. In a large scale insect farm there could be many hundreds of growing enclosures. At such a scale the individual monitoring of growing enclosures will be less than practical. Therefore, an automated surveillance system which can provide essential information is needed. Such information could be: the number of insects, sex balance of the population, and size of individual insects within an enclosure, along with environmental conditions such as temperature, humidity, and CO_2_ levels. This information from the system could be used to calculate other information such as activity/movement levels, amount of food remaining in an enclosure, and signs of pathogens/predators, to provide an autonomous closed-loop control system for each enclosure and also to provide management information direct to farmers.

In addition, to these somewhat obvious measures that can be leveraged cheaply from off the shelf IoT devices, the development of advanced instance level segmentation and identification algorithms, may allow us to begin to monitor individual inter-actions of insects at a level that has been prohibitive in the past, such as those identified in (5). In traditional livestock farming, we are seeing the emergence of long term identification and tracking systems. Example include pig farming (6) and in dairy herd management (7), allowing social networks to be analysed and more fine-grained approaches to welfare adopted. It is not beyond imagination that similar techniques can be applied to insect populations to bring about a deeper knowledge and appreciation of colonies, as well as being able to provide enclosure level information concerning the welfare of its inhabitants based on individual behaviours.

This article presents results from two pilot experiments involving BSF and domestic crickets. We report on the acquisition device hardware which is designed to be low cost, that when combined with state-of-the-art deep learning techniques allow us to count, size, and sex the insects. Although important previous work in the area of insect classification has been carried out by: Hoye et al. (8), Valan et al. (9), Blair et al. (10), Hansen et al. (11), there has been little prior work in the specifics of insect detection and sex classification, less on using real world images, and no known work for sex detection using real world images. Our motivation for focusing on size classification for BSF is in order.

## 2. Materials and Methods

This section is broken into two subsections describing our work using machine vision on BSF and domestic crickets (*Acheta domesticus*), the first to measure the size of BSF larvae, the second to count and sex crickets. All machine vision code and data analysis software was written in Python 3.8 using publicly available libraries (Tensorflow, NumPy, scikit-learn).

### 2.1. BSF

There is a tendency of BSF farmers to favour larger flies as they have a higher reproduction rate, the larvae are bigger and thus contain more protein than smaller larvae. Manual selection of such traits at an industrial level is currently infeasible due to the high numbers of larvae making it economically unviable. We therefore look at the first stage of automating this process by training an object detector to distinguish those larvae that are larger than a given threshold and are therefore suitable for breeding, and those that are smaller and should be euthanised for protein.

The whole process of measuring, image capturing, and recording the sizes for the creation of this initial dataset was manual. The focus of this work was to validate that large pupa can be identified for selective breeding rather than precision measurement. Using machine vision for measuring objects is a classic use case, but often fraught with issues, such as lens distortion, occlusion, shadowing, etc. in unconstrained capture environments. As the larvae are non-rigid, the system needs to be able to also cope with this, so an overall deep-learning based classification system has been developed rather than a measurement tool. As such, the experiments have not been replicated (as might be expected in biological studies) – the larvae were grown, imaged and measured. Future work will commence the important work of investigating the effects of climate on their behaviour and having control experiments in place to allow statistical analysis through replication studies. Future work will focus on investigating whether it is also possible to determine the sex of the pupa reliably using machine vision, something which is currently extremely difficult to do manually. This is discussed further in Section 4.

#### 2.1.1. Acquisition

**Figure 1:**
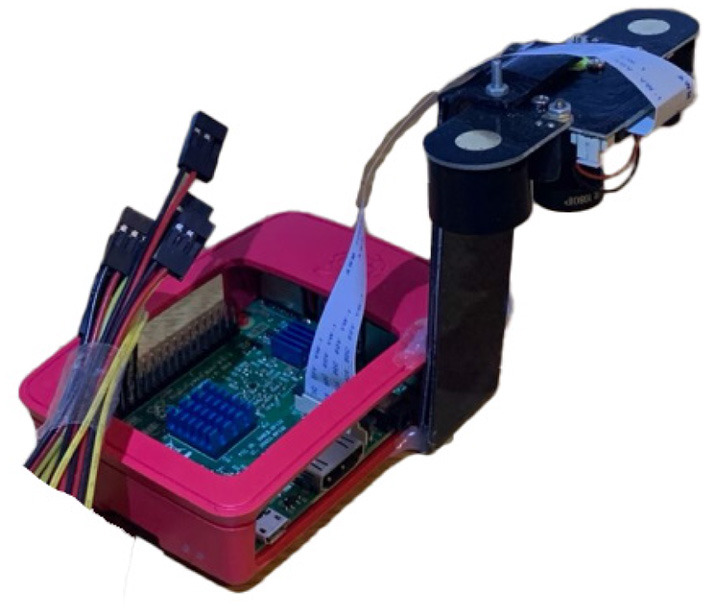
Device for capturing images of BSF larvae—ASHATA IR camera module, connected to a Raspberry Pi 3 B+.

Figure 1 shows the equipment used to capture images of BSF larvae. BSF larvae are negatively phototactic (i.e., they avoid strong sources of light) (12). Therefore, in order to image continuously over 24 h, Near Infra-Red (NIR) light sources (850 nm) were used to illuminate the environment without disturbing the larvae. This is an important consideration for any commercial implementation and here while we use the NIR images for analysis, the visible light source images could be used for sorting dried BSF pupae.

As there is no publicly available Black Soldier Fly dataset, a BSF colony was farmed to acquire images to train an object detection model. Thus, 500 BSF larvae were purchased to populate the colony. The BSF were kept at 27.6^*o*^C temperature, 60–65% relative humidity, and fed a diet consisting of fresh vegetables, fruits, and plant-based products. The pre-pupae were collected daily from the storage section and placed in a location with controlled ambient illuminations (i.e., out of direct sunlight to ensure consistent illumination) to capture their images. An “ASHATA” IR camera module connected to a “Raspberry Pi 3 B+” was used to capture images. The camera was mounted at the height of 9 cm, which was empirically determined to ensure that the pre-pupae were of suitable size in the images. Images were captured with the maximum resolution possible, which was 5 MP (2592 x 1944 pixels). In total, 310 visible light and 310 NIR images of individual larvae were captured and used for classification experiments.

The object detection model chosen for deployment was an SSD-MobilenetV2 with an image resolution of 300 x 300 pixels as this lightweight model is capable of running on low power edge devices such as a Raspberry Pi.

A Train:Test:Validation split of 60:20:20 was used.

### 2.2. Crickets

#### 2.2.1. Acquisition

**Figure 2:**
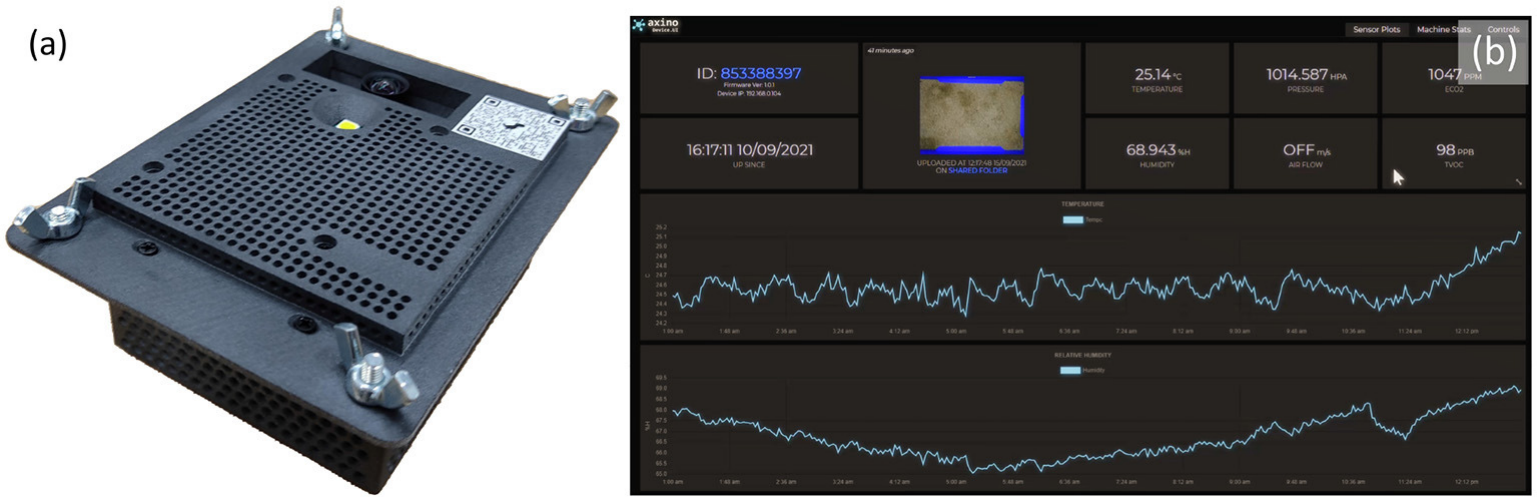
**(a)** The “Insecto” device providing temperature, humidity, CO_2_, air pressure, and volatile organic compound levels environmental condition information together with timelapsed image capture that was used to capture the images of crickets from the University of Calabar. **(b)** Screenshot of the data captured and displayed from a webservice.

Images were captured from a cricket growing facility in Calabar, Nigeria where researchers from the University of Calabar have been carrying out work into the commercial growing of crickets (*Acheta domesticus*) for agricultural purposes. An Insecto device, a system developed by SciFlair, Bristol, specifically for the monitoring of cricket enclosures, was used to capture these images. The device is equipped with a range of sensors for measuring: humidity, air pressure, CO_2_, and volatile organic compounds (VOC); as well as a camera module, LED lamp, and internet connectivity. The device was mounted to the roof of one of the growing vessels so that the camera captured a top down image of the enclosure. A view of the bottom of the Insecto device is shown in Figure 2. An example of one of the images captured by the Insecto device can be seen in **Figure 5**. The Insecto device was configured to capture an image each hour it was powered on. These images were then uploaded to an internet cloud storage service. This image is used as input to the YOLOv5 model. The camera module used in the Insecto device is a Raspberry Pi Camera with a Sony IMX477 CMOS sensor. Each image captured was a 24 bit sRGB 4056 x 3040 pixel jpeg image.

In the period starting from the 12th of July until the 8th of August 2021, 195 images were uploaded to the cloud storage service. Of these 195 images a random sample of 100 images were selected in which the crickets were marked with bounding boxes using the Computer Vision Annotation Tool (CVAT), a free open source tool developed and made available by Intel (13). In total, 2,796 crickets were labelled. These labels were used to train the YOLOv5 object detector to count the insects.

**Figure 3:**
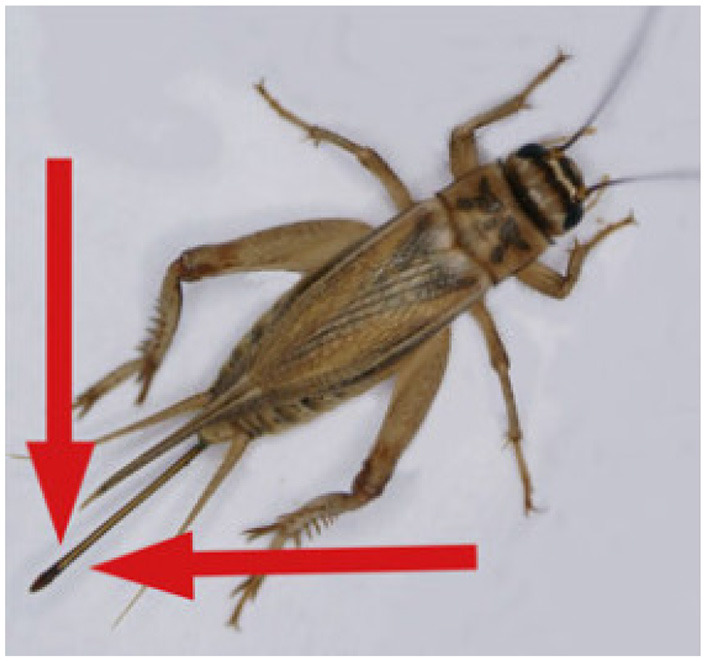
An adult female Cricket, *Acheta domesticus*. Identifiable as female due to ovipositor (marked by red arrows). Image modified from (14).

The second stage, classifying the sex of the cricket, was performed by adding additional annotations to these bounding boxes based on the visible presence of an ovipositor as seen in Figure 3. It should be noted that crickets which received a “Female” attribute with a “False” value likely included some images of females with occluded ovipositors, females which were too blurry to positively identify their ovipositors, and juvenile females/Nymphs with undeveloped ovipositors. Crickets without an ovipositor visible therefore cannot definitively be identified as male due to the presence of these immature female, occluded, and blurry instances. For this reason a Female[True/False] class was selected over Female/Male classes for the cricket annotation.

For the actual sex classification, feature extraction was performed using VGG-16 (15) with pre-trained ImageNet weights, and the outputs from the final convolutional layers are then used in an SVM for classification.

Again, a Train:Test:Validation split of 60:20:20 was used. The test dataset (20% of total dataset) contains 20 images taken from the cricket growing container with 268 cricket instances (100 female, 168 male) which were manually annotated.

We present our validation results in Table 1 in terms of precision [Equation (1) what proportion of positive identifications were actually correct]; recall [Equation (2) what proportion of actual positives were identified correctly]; and F1 [Equation (3) the harmonic mean of the two which provides an additional measure of the accuracy]. The third row in Table 1, “Accuracy” presents the overall accuracy, i.e., the number of correct identifications out of the total number of images.


(1) $$\begin{array}{r} Precision=\frac{\text{True Positive}}{\text{True Positive}+\text{False Positive}} \end{array}$$

(2) $$\begin{array}{r} Recall=\frac{\text{True Positive}}{\text{True Positive}+\text{False Negative}} \end{array}$$

(3) $$\begin{array}{r} F_{1}=2\times \frac{\text{Precision}\times \text{Recall}}{\text{Precision}+\text{Recall}} \end{array}$$

**Table 1:** VGG-16 sex classifier results on the Calabar dataset.

	**Precision**	**Recall**	**F1**	**N**
Female	0.91	0.91	0.91	98
Unknown	0.95	0.95	0.95	164
Accuracy	-	-	0.93	262
Macro Avg	0.93	0.93	0.93	262
Weighted Avg	0.93	0.93	0.93	262

*Metrics are defined by Equations (1)–(3). N is the number of observations these are based on*.

## 3. Results

Here, we present the results from our pilot studies for measuring the size of BSF larvae, distinguishing whether the larva is best suited to breeding or consumption, as well as counting and sexing crickets.

### 3.1. BSF

**Figure 4:**
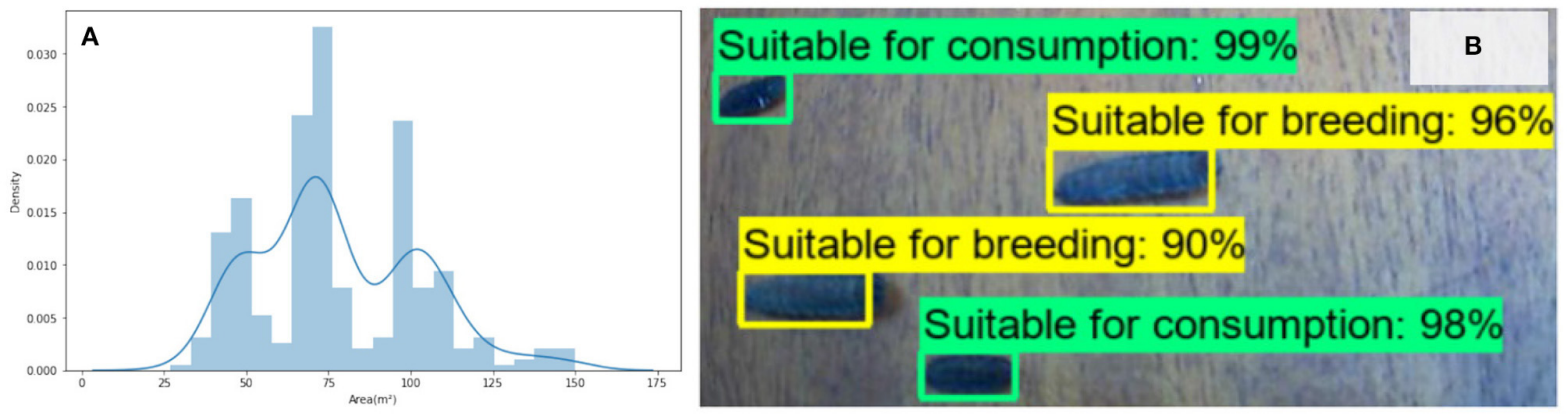
**(A)** The tri-modal distribution of BSF larvae size. **(B)** Example image of BSF classification trained primarily on size, and confidence levels with mAP score of 0.87.

The size distribution of the BSF shown in Figure 4. An example output from the trained SSD-MobilnetV2 model is also shown in Figure 4. The trained model performs with a Mean Average Precision (mAP) of 0.87 which indicates that it is able to correctly classify the larvae in 87% of cases in the validation set.

### 3.2. Cricket Detection and Sexing

**Figure 5:**
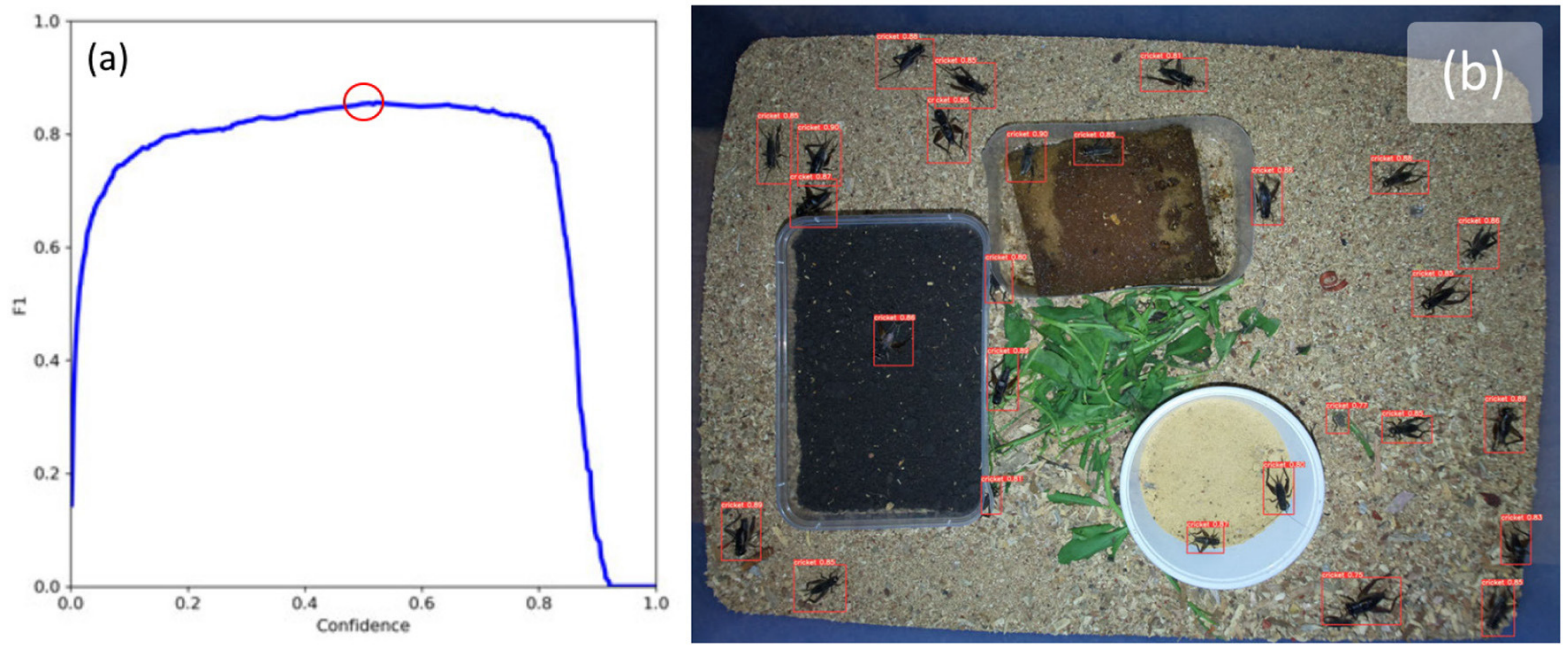
**(a)** F1 confidence curve shows best F1 score of 0.86 with a confidence threshold of 0.523 (highlighted with red circle), and **(b)** example results of cricket detection using YoloV5 with confidence threshold set to 0.523.

Figure 5b shows an example image with bounding boxes and confidence intervals overlaid after running inference with the trained model. The model was able to detect crickets with an F1 score of 86% when the confidence threshold was set to 0.525 (F1 vs. confidence can be seen in Figure 5a).

**Figure 6:**
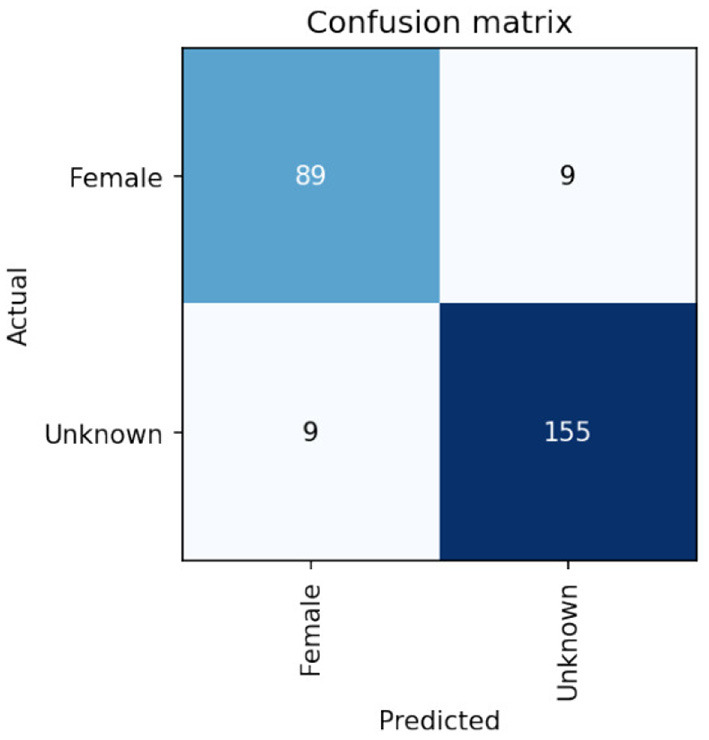
Confusion matrix of sex classification from cropped detections using transfer learning based on VGG-16 base model weights showing 89 out of 96 females are correctly identified.

On the Calabar test set the VGG-16 network achieved very promising performance after 30 epochs of training. Results were achieved which gave an F1 score of 93% for the sex classification of the crickets as shown in the performance breakdown by class/sex in Table 1. The confusion matrix showing the comparison of Actual/ground truth data against the predicted class results is shown in Figure 6. Some discussion of the implications of these results can be seen in the next section.

## 4. Discussion

In summary, our results show the following:

1. High accuracy detection of live *Acheta domesticus* with 86% F1 score at 0.523 using YOLOv5.
2. High accuracy classification of real world *Acheta domesticus* to a sex level by further training a pre-trained convolutional neural network. Achieved F1 scores of 93% using VGG-16 with an SVM.
3. High accuracy detection of BSF larvae of 87% mAP using SSD-MobilenetV2.
4. Initial successful trials of the “Insecto” device to remotely gather images and environmental information using a low cost device with minimal setup and maintenance requirements.

This research shows that the application of deep learning models to both the detection of *Acheta domesticus* and classification of their sex is accurate enough to be useful for commercial cricket growing organizations. These results also have promise for deployment in camera traps to detect and classify other species to a species as well as classify sex from real world images.

While the accuracy of the cricket detector is reasonable at 86%, it can be seen in Figure 5b that some highly occluded crickets were identified by the detector and all non-occluded crickets were also identified. However, three instances of detection errors can be observed in this image which are indicative of the models performance on the rest of the dataset. The first error is a false positive with a confidence of 0.77 which is located to the right of the white container, the second is a pair of crickets enclosed by a single bounding box to the bottom right of the white container, and the third is a highly occluded cricket near the top region of the green leaves. These three instances represent the typical situations where the model fails: when crickets are highly occluded, when crickets are overlapping or on top of one-another, and where there are objects which have features which appear vaguely cricket-like and are next to green objects; it seems that the model confuses these with highly occluded crickets.

It is likely that further improvements can be made by optimising the chosen architecture as well as providing larger amounts of training data. Typically, the errors result from highly occluded or tightly clustered/overlapping insects. A similar issue is described and mitigated by using a modified Mask-RCNN (16) for separating tightly clustered pigs by Tu et al. (17). The suggested approach should generalise well to crickets.

With regards to BSF larvae, our results demonstrate the feasibility of being able to separate colonies based on phenotypic traits (in this case size) with 87% accuracy. With a larger dataset, the performance of this is likely to be improved upon. It is hypothesised that this method causes the next generation to favour larger sized larvae. We, therefore, suggest that further work compares the generational effects of selective breeding through the automation of this process. It is therefore proposed that future work should perform a controlled group experiment, to acquire two similar BSF colonies and perform selective breeding on one of the colonies over a number of generations. Except for this, all the remaining conditions must be the same. Only then, the resulting performance plots could be compared to conclude the approach's effectiveness.

Although a possible product of a relatively small sample size, it is interesting that a tri-modal distribution is shown in the sizes of the larvae in Figure 4A. While the overall distribution is normal, the clear troughs either side of the modal peak are unexpected, and the reason for the outer peaks is not known but does infer that if this distribution is replicated across other colonies, and that these phenotypes are inheritable, then automated systems that rely on vision based sizing will be able to identify and separate individuals based on these size distributions. While it might be expected that the sizes would fall under a normal distribution, or that as suggested by Putra and Safa'at (18), that female pupae are larger, that a bi-modal distribution (corresponding to the sex), the presence of the central peak indicates other influences (perhaps based on conversion performance) are present.

While environmental data was captured successfully by the Insecto boxes in Nigeria, and the robustness of the device demonstrated, the actual data has not yet been analysed nor incorporated into any control loop. Future work will seek to automatically adjust local temperatures, humidity, and airflow in order to assess their impact on the colonies and develop optimal conditions for the insects to thrive. This type of low-cost, low-maintenance device could prove to be exceptionally useful for smallholder farms to monitor small scale insect farms that could provide a reliable source of protein for livestock in developing countries. It could also be used in large scale farms to remotely monitor conditions where manual inspection would be prohibitively time consuming. Future work will use the environmental monitoring data to provide a closed loop feedback system to maintain conditions, and be used to determine optimal parameters to increase yields in comparison to control groups which are controlled at the room level.

With minimal effort, it will also be possible to detect anomalous behaviour which may offer early indications of problems in a colony, for example disease which requires intervention. Using the object detection frameworks presented here, it would also be possible to assess whether an insect has not moved for a prolonged period of time and, if it is an isolated case, remove it from the tray or alternatively dispose of the whole tray if many such incidents are detected.

It should be acknowledged that there are limitations associated with this early work. For example, the sex classifier has only been tested on one species of cricket under relatively controlled conditions. This species has very visible sex characteristics, i.e., a large ovipositor. This means that the same level of accuracy is less likely for other species with less visible sex characteristics. It would be interesting to investigate the possibility of sexing BSF larvae using machine vision. While it is possible to sex adult BSF there is only limited work on attempting to sex pupae. Putra and Safa'at (18) showed that there was a significant correlation between the length of pupae and the sex (the longer the pupa, the more likely it was to be female) and reported classification accuracy of 62%. Through a longitudinal study it would be possible to generate a dataset of pupa that resulted in male or female BSF and then use a CNN in an attempt to extract more subtle features than the pupa length and increase the predictive accuracy. If this is found to be the case, then care must be taken when selecting individual larvae for subsequent generations, that sufficient numbers of the smaller males are included for viability. This also justifies the need to investigate more robust indicators of sex in BSF larvae rather than their size alone.

However, both experiments clearly show that machine vision can be used for counting, sizing and sexing insects reliably in typical insect farming environments and pave the way for a great deal of future work in which more complex features such as inter-actions (e.g., aggression or mating events) might be detected and recorded. This represents a considerable step forward towards automating such processes. It is possible to envisage such systems being able to guide robotic arms, perhaps with soft-robotic end-effectors to pick and place individuals between different colonies to balance overall numbers and ensure a balance of males and females as well as selection of individuals with desired phenotypes for future generations.

## 5. Conclusions

We demonstrate the efficacy of object detection and classification methods on two types of insects commonly farmed as sources of protein. We show that machine vision can be used for accurately counting, sizing, and sexing (in the case of crickets), where this important information can be used to effectively monitor colony health and potentially assist in automatically selecting desirable traits for future generations. This paves the way for further work in automated closed-loop insect farming and in exploring the ability to monitor insect behaviour at colony, and potentially individual levels.

## Data Availability Statement

The datasets presented in this article are not readily available due to commercial sensitivities. Requests to access the datasets should be directed to mark.hansen@uwe.ac.uk.

## Author Contributions

MH corresponding author and PI of GCRF project, collated and oversaw project, and experimentation. AO cricket rearing and facilities, insect consultant, and wrote sections of the manuscript. RG cricket image analysis and wrote sections of manuscript. AK BSF rearing, image analysis and wrote sections of manuscript. MS Manuscript review. FT design and build of Insecto device and manuscript review.

## Funding

This work was partly funded by the GCRF Agri-tech Catalyst Seeding Award managed by Biotechnology and Biological Sciences Research Council, UK Research and Innovation, (UKRI-BBSRC). Award Ref: GCRF-SA-2020-UWE.

## Conflict of Interest

FT was employed by the company SciFlair Ltd. The remaining authors declare that the research was conducted in the absence of any commercial or financial relationships that could be construed as a potential conflict of interest.

## Publisher's Note

All claims expressed in this article are solely those of the authors and do not necessarily represent those of their affiliated organizations, or those of the publisher, the editors and the reviewers. Any product that may be evaluated in this article, or claim that may be made by its manufacturer, is not guaranteed or endorsed by the publisher.
